# Plant Cell and Organism Development

**DOI:** 10.3390/ijms21165636

**Published:** 2020-08-06

**Authors:** Robert Hasterok, Alexander Betekhtin

**Affiliations:** Plant Cytogenetics and Molecular Biology Group, Institute of Biology, Biotechnology and Environmental Protection, Faculty of Natural Sciences, University of Silesia in Katowice, 40-032 Katowice, Poland

Plants represent a unique and fascinating group of living organisms. By utilising photosynthesis, they are primary producers, which in one way or another are indispensable to the existence of heterotrophs, including humans. They also absorb carbon dioxide and release oxygen into the atmosphere, which is crucial to sustain not only the Earth’s biosphere but also the geosphere. As sessile organisms, plants had to develop unique strategies to deal with environmental stresses. These involve, inter alia, the presence of the cell wall, plant cell totipotency and a predisposition of plants to panorganismal polyploidisation, including interspecific or even intergeneric hybridisation followed by a stable restoration of sexual reproduction via allopolyploidisation.

These and other compelling phenomena, which have been studied in both weedy model plants such as *Arabidopsis thaliana* (Arabidopsis) and *Brachypodium distachyon* (Brachypodium) and in various crop species, were the sources of our inspiration to conceive the Special Issue “Plant Cell and Organism Development”. The issue consists of 38 peer-reviewed papers; 29 articles and nine reviews.

In the first article, Gorpenchenko et al. [1] present a comprehensive analysis of the tempo-spatial patterns of the aporphine alkaloid stepharine accumulation in the morphogenic tissues of *Stephania glabra*. They performed a combination of the morphological and histological analyses of various cell lines using both light microscopy and scanning electron microscopy, followed by mass spectrometry to determine the stepharine content and spatial distribution within the defined structures of microdissected tissue. They found that the morphogenic cell cultures of *S. glabra* not only produce large amounts of stepharine, but also allow micro-propagated plants to be obtained in large numbers. Their research reinforces our understanding of the alkaloid distribution and can be helpful in developing new plant cell cultures for the stable production of aporphine alkaloids.

In their article, using light microscopy and transmission electron microscopy, Milewska-Hendel et al. [2] analysed the uptake of gold nanoparticles (AuNPs) by the root cells and protoplasts of Arabidopsis. They revealed that regardless of their surface charge, AuNPs did not cross the cell wall in Arabidopsis roots. However, they observed that the AuNPs that had a different surface charge caused diverse changes in the root histology and root cell ultrastructure. Using the protoplasts as a model system enabled them to determine the role of both the cell wall and plasma membrane in the AuNP uptake. Their study is of importance in the context of the ever-growing industrial use of AuNPs, which results in their release into the environment.

The review by Pinski et al. [3] discusses the current state of knowledge about the genetic basis of the interactions between plants and endophytic bacteria. They focus on various genetic features of the endophytic bacteria that are involved in interactions with plants and vice versa, i.e., the genetic features of plants that are behind their interactions with endophytic bacteria. One of the key conclusions is that gaining a better understanding of such interactions can be crucial for the successful inoculation of plants with growth-promoting endophytes, which can lead to a measurable increase in crop health and yield.

In another review, Kumar et al. [4] focus on the *INDETERMINATE DOMAIN* (*IDD*) genes that encode zinc finger proteins, which act as transcription factors that regulate various developmental and physiological processes that are evolutionarily conserved across the plant kingdom. They discuss the structure and phylogenetic analysis of these proteins, followed by their biological functions, which are linked with the regulation and modulation of sugar metabolism, floral transition, starch accumulation, cold response, seed development, shoot gravitropism, secondary cell wall formation, hormonal signalling, ammonium uptake and overall plant architecture. By considering such an all-embracing impact of the IDD proteins on plant growth and development, the authors conclude that gaining a better understanding of their function will be of great importance for genetic programmes in terms of the signalling networks that are linked with the regulation of plant development and metabolism, which are connected with external environmental fluctuations.

In their article, Jaskowiak et al. [5] show how aluminium alters the root architecture and pectin cell wall composition in barley, which is one of the key temperate cereals. Aluminium is known to be one of the most important crust elements that cause decreased plant production in acidic soils. Using histological and immunohistochemical analyses, they concluded that aluminium is responsible for the tissue- and cell-specific changes in both the composition and localisation of some pectic epitopes in barley roots. Since these epitopes are known to be involved in processes such as maintaining cell wall flexibility and stiffening as well as firming the cells, this may indicate that they may be responsible for changes in the physiological properties of the cell wall response to some stressors, for example, the soil pH and its aluminium content.

The article by Han et al. [6] presents the role of *RcAP1*, which is the homologue of the *APETALA1* (*AP1*) gene in *Rosa chinensis*. Together with some other MADS-box genes, it encodes the protein that acts as a transcription factor that is essential for the formation of the floral meristem and floral organs. Though the functions of the MADS-box genes have been extensively studied in Arabidopsis, their functional analysis in the China rose is limited. The authors found that the overexpression of *RcAP1* in Arabidopsis induced early flowering, but that its downregulation in *R. canina* delayed flowering. Considering that the expression of this gene was specific to the sepals of the floral organs and was downregulated in the deformed sepals and leaf-like organs, they concluded that *RcAP1* may influence rose organ floral morphogenesis. These results may not only provide a new insight into the regulatory mechanisms of the rose floral organs but may also be useful for the comprehensive analyses of flower opening in this important ornamental plant.

In another article, Kong et al. [7] provide a functional analysis of the *INDOLE-3-BUTYRIC ACID RESPONSE 5* (*IBR5*) gene that encodes a dual-specificity phosphatase, which is a key component of the leaf-serration machinery. By studying Arabidopsis loss-of-function mutants for *IBR5*, they revealed that pronounced serrations are caused by an increased cell area. The introduction of a C129S mutation into the highly conserved motif of IBR5 made this protein unable to rescue the leaf-serration defects of the *ibr5-3* mutant. These and the other results that are presented in this article suggest that IBR5 is critically important for regulating the leaf serration development by altering the auxin efflux protein PIN1-directed auxin maxima distribution.

In their article, Wang et al. [8] focus on *APETALA3*, which is another representative of the MADS-box genes that are crucial for the development of floral organs. In their attempt to elucidate the molecular mechanisms that are behind the morphological defects in transgenic plants, they revealed that in Arabidopsis, it is the exogenous *AP3* promoter that is responsible for the *APETALA3* silencing, which leads to the occurrence of *ap3*-like phenotypes. Interestingly, the occurrence of such phenotypes turned out to be correlated with the DNA hypermethylation of the *AP3* promoter. Since the expression of *AP3* was reactivated and the methylation of the *AP3* promoter was reduced in the RNA-directed DNA methylation pathway-defective *AP3*-silencing lines, it seems that this methylation pathway is responsible for the transcriptional silencing in these transgenic lines. The general conclusion is that it may be common in transgenic plants that the exogenous promoter fragments can trigger the methylation of the homologous endogenous sequences.

The article by Awada et al. [9] concerns the first global analysis of the somatic embryogenesis (SE) metabolomics and hormone dynamics, and attempts to elucidate the mechanisms that regulate cell fate and totipotency in coffee (*Coffea arabica*), which is not only one of the key commodity crops but is also one of the tree model species. They performed the detailed analyses of more than 100 metabolites and revealed that a massive re-configuration of metabolic pathways induced SE. This study helped to divide the 14 sampled stages of the SE process into five important phases, which led to the identification of four key developmental phase switches that are crucial for the entire biological efficiency of embryo regeneration. The results should not only be useful for coffee micropropagation but also for culturing many other economically important plant species.

The review by Borek et al. [10] provides a comprehensive and up-to-date examination of plant peroxisomes and their diverse and versatile functions, with particular attention being paid to their degradation via autophagy. They introduce the reader to different kinds of autophagy such as macroautophagy and microautophagy, and then focus specifically on the various forms of pexophagy, which is a peroxisome-specific form of autophagy. The authors conclude that although pexophagy also exists in other eukaryotes, it is likely that there is a specific mechanism for the selective degradation of peroxisomes in plants.

The article by Valencia-Lozano [11] is another paper on coffee (*Coffea arabica*). It demonstrates an efficient protocol that enables the particle bombardment-mediated stable genetic transformation of coffee plants that express the Cry10Aa protein of *Bacillus thuringiensis* (Bt) for the first time. There are many Bt-originated sequences encoding the insecticidal Cry proteins, which can be transferred and expressed in genetically modified plants. Such a strategy, when successful, provides protection against parasitic insects and reduces the necessity for applying a synthetic pesticide. However, coffee belongs to the plants that are not easily transformable. The protocol that was developed in this work emphasises the importance of manipulating the gelrite-induced osmotic stress response of the genes that are involved in somatic embryo maturation for the efficient transformation with *cry10Aa*, the expression of which in coffee plants creates the possibility to better control the coffee berry borer.

In the following article, Lin et al. [12] characterise the *Spike Activator 1* (*SPK1*) gene that was isolated from one of the moth orchids, *Phalaenopsis aphrodite*, which is among the most important ornamental plants in the world. *SPK1* is the member of the basic helix-loop-helix gene family, which encode the transcription factors that are involved in various aspects of plant physiology. Using virus-induced gene silencing, the authors demonstrated the importance of the SPK1 protein as a regulator that mediates processes such as axillary bud development, spike initiation and reproductive organ development in the moth orchid. Gaining a better understanding of the spiking regulation in *P. aphrodite* could have some practical importance for the global floriculture industry.

The article by Yu et al. [13] provides an efficient, mature-embryo-derived *Agrobacterium*-mediated transformation system for the flagship temperate-zone model grass, Brachypodium, reference line Bd21. Although several transformation systems have already been well established for this species and line, they usually utilise immature embryos, which have a better regeneration capacity. The authors also reported the effectiveness of a newly developed chemical inducer, Fipexide, to induce callus, roots and, in particular, shoots, which were generated in the mature embryo-based system more efficiently than with the chemicals that are routinely used for this purpose, such as kinetin and thidiaruzon. It is possible that this transformation system will provide a considerable advantage when transforming other inbred lines and wild accessions of Brachypodium, and perhaps, even other *Brachypodium* species, which are more recalcitrant to other transformation protocols. This, in turn, could advance the use of *Brachypodium* as a model genus in grass genomics.

In another article, Meidani et al. [14] characterise the modifications in the call wall composition in the so-called giant cells (GC) that are induced by *Meloidogyne incognita*. This root-knot nematode is an obligatory plant parasite and is one of the most notoriously unmanageable crop pests with a wide host range. In their study of GCs in the wild-type Col-0 ecotype of Arabidopsis and the microtubule-defective *fra2* katanin mutant, both of which had been infected with the parasite, the authors revealed that the mutant was more susceptible to the infection. They also observed that the microtubule (MT) katanin severing defects of the *fra2* mutants led to an altered pectin and hemicellulose distribution in wild type GC cell wall, compared to the wild type. They conclude, inter alia, that katanin MT severing may be an important but insufficient element in the plant defence against this parasite.

In their article, Godel-Jędrychowska et al. [15] describe the impact of phytosulfokine (PSK), which is a peptidyl plant growth factor that acts as an intercellular signalling molecule and is involved in cellular proliferation and dedifferentiation on the cell wall regeneration in protoplast cultures of three *Daucus* taxa. Using the immunolocalisation of several antibodies that target various pectic, arabinogalactan protein and extensin cell wall epitopes, they observed that PSK induces diverse responses of the *Daucus* taxa to PSK with regard to the protoplast-derived cell development and diverse chemical compositions of their reconstituted cell walls. These findings enhance our understanding of the role of the chemical constitution of the cell wall in the plant differentiation processes, which can contribute to suppressing protoplast recalcitrance. This, in turn, could be beneficial to crop improvement.

The article by Stawska and Oracz [16] focuses on the involvement of blue light (BL) in the seed biology of dicotyledonous plants. Using Arabidopsis, they addressed several crucial research problems regarding seed dormancy and/or germination and the possible associations between the BL action and the involvement of specific elements of the light signalling pathway and some of the phytohormone (GA, ABA) signalling pathways. Based on the results of a functional analysis of several T-DNA insertion mutants and the overexpression of transformants in the *HY5*, *HFR1*, *HYH* and *LAF1* genes that encode the transcription factors, they propose a hypothetical model of BL action in regulating the alleviation of dormancy and the stimulation of the germination potential of dormant Arabidopsis seeds.

In the following article, Baba et al. [17] describe how the calcium-dependent protein kinase-related kinase 5 (CRK5) contributes to the progression of embryogenesis in Arabidopsis. In their previous study, the authors demonstrated that AtCRK5 can directly or indirectly regulate some of the polar auxin transport PIN proteins. Using various experimental approaches, in this study, they revealed that AtCRK5 also has the capacity to phosphorylate the hydrophilic loops of other embryogenesis-related PIN proteins in vitro. They conclude with a model which claims that AtCRK5 can also govern the embryo development in Arabidopsis, by fine tuning the auxin-GA levels via the stability/abundance of the polar auxin transport proteins, in addition to its regulatory role in the root gravitropic responses and hypocotyl bending.

The review by Tripathi et al. [18] describes how the plant phytohormones regulate photomorphogenic development. After a general introduction to the importance of light and phytohormones in various aspects of plant existence, they discussed the role of phytohormones in regulating the key developmental stages in the plant life cycle in detail, i.e., seedling establishment and determining the plant architecture, followed by the effect of light quality and quantity on plant vegetative growth, flowering and finally senescence. In their concluding remarks, the authors highlighted the differences in the phytochrome functional diversity between dicots and monocots stressing the need to better understand phytochrome-mediated light signalling and photomorphogenic development in monocots as being imperative in order to improve crops in light of global warming due to climate changes.

The article by Jun et al. [19] focuses on revealing the still unclear molecular basis of the regulatory gene networking during leaf development. The authors studied the spatiotemporal patterns of the *ORESARA15* (*ORE*) expression. It is already known that this gene encodes one of the plant A/T-rich sequence and zinc-binding proteins (PLATZ), which act as transcription regulators and enhances leaf growth. In this study, using the loss of function and gain-of-function mutants of *ORE15*, they characterised cell proliferation during leaf growth in Arabidopsis. The successful generation of mutants for some of the other cell proliferation-related genes, such as *ANGUSTIFOLIA3*/*GRF-INTERACTING FACTOR1* (*AN3*/*GIF1*) and *AINTEGUMENTA* (*ANT*), enabled them to perform genetic and anatomical analyses, which resulted in them proposing a working model of the interactions between these genes in the cell proliferation regulatory pathway during leaf growth.

In the following article, Ajadi et al. [20] report the biological functions of two rice KRPs (kip-related proteins), KRP1 and KRP2. KRPs are cyclin-dependent kinase inhibitors that are involved in controlling the plant cell cycle and modulating various developmental processes. In this extensive study, the authors, inter alia, phenotypically characterised the *KRP1* overexpression lines, the *krp2* single mutant and the *krp1*/*krp2* double mutant, and revealed that all of them displayed poor seed production, delayed seed germination and impaired early seedling growth. These and other findings are a strong indication that both of these KRPs are important for seed morphogenesis. They also demonstrated that KRP1 interacted with two cyclin-dependent kinases, CDKC;2 and CDKF;3, thus indicating their possible involvement in the KRP1-mediated seed development.

In another article, Cui et al. [21] did a genome-wide characterisation of the 158 genes encoding the calcineurin B-like protein-interacting protein kinases (CIPKs) in two cotton species, *Gossypium hirsutum* and *G. barbadense*. These kinases are among the key regulators of plant growth, development and response to various stresses. This study provides a comprehensive analysis of the identified *CIPK* genes for a phylogenetic classification, gene structure, protein motifs, chromosomal locations and duplicated genes, which is followed by a comparison of the expression profiles of the *GhCIPK* genes in different tissues and under various abiotic stresses as well as the co-localisation of these genes with the QTLs for the seed oil and protein content in the world’s number one fibre plant.

The article by Zhang et al. [22] presents a comprehensive analysis of the tubby-like protein (*TLP*) genes in tomato. TLPs are ubiquitous in eukaryotes; however, while their functions in animals are well studied, relatively little is known about their role in plants. Using a genome-wide identification and phylogenetic analysis, the authors revealed 11 *TLP* gene families and classified them into three subgroups. Using the qRT-PCR expression profiling of the *TLP* genes in different tomato tissues of the wild-type plants, some ripening mutants and after exogenous ethylene treatments, they found that seven *TLP* gene families were likely to contribute to various aspects of vegetative and generative plant development. Interestingly, four other *TLP* gene families seem to be specifically involved in the fruit ripening of this second most important vegetable crop in the world.

In the following article, Jia et al. [23] identified a novel gene in *Glycine soja* (wild soybean), *Gs5PTase8*, which encodes one of the inositol polyphosphate 5-phosphatases (5PTases) that are common in plants and that participate in various processes of their development and stress response. In their comprehensive study, the authors, inter alia, demonstrated that Gs5PTase8 is localised in both the protoplast and the apoplast. They also found that the ectopic expression of *Gs5PTase8* significantly increased salt tolerance, not only in the soybean hairy roots, but also in transgenic Arabidopsis plants and tobacco cells, which makes this gene a promising candidate for improving soybean adaptation to salt stress.

The review by Betekhtin et al. [24] provides an extensive overview of the uses and challenges of in vitro tissue cultures in the *Brachypodium* genus. They discuss callus induction in this model genus, followed by various methods for genetic transformation, protoplast-based assays, the use of *Brachypodium* transformation in functional genetics and gene editing in *Brachypodium*. The last part of this review focuses on the various factors that make grass species be recalcitrant in a tissue culture and/or genetic transformation.

In another review, Wojcik et al. [25] describe the recent advances in studies on the auxin-controlled genetic network that controls the induction of somatic embryogenesis (SE) in plants with particular attention being paid to Arabidopsis. After introducing the reader to the basics regarding SE, the authors reviewed the main SE inducers, such as auxin and its synthetic mimicker 2,4-D and discussed auxin biosynthesis and accumulation during SE. In what followed, they focused on the core regulatory components of the auxin-signalling pathways that are involved in SE and the complex interactions between the transcription factor genes that control auxin-induced SE. The conclusion of this review indicates the importance of identifying the epigenetic processes that are involved in regulating SE.

In their article, Xiao et al. [26] reveal the mechanisms of cellulose and pectin polysaccharide metabolism in fibre cell walls during the tension wood (TW) formation in *Catalpa bungei*, by combining transcriptomic and proteomic approaches as well as using Raman spectroscopy. The authors found, inter alia, that there was no obvious gelatinous layer in the TW of this species and that the secondary wall depositions in the TW were fewer compared with other kinds of wood. They conclude that their results will provide an enhanced insight into the TW formation in other important timber tree species, and will contribute to gaining a better understanding of the mechanisms that are behind xylogenesis and TW formation. This could aid the future genetic improvement of wood properties.

In the following article, Sirl et al. [27] describe the function of *AHL18* in Arabidopsis. This is a novel member of the *AT-hook Motif Nuclear Localised Protein* (*AHL*) gene family whose representatives are known to modulate gene expression and regulate various biological processes in land plants. By analysing the *ahl18* knock out mutant and *AHL18* overexpression lines, the authors identify the role of this gene in the formation of the early root system architecture due to its impact on the length of the proliferation domain and the number of dividing cells in the root apical meristem (RAM). The authors postulate a clear regulatory function of *AHL18* in the RAM activity, onset of differentiation, initiation of lateral root primordia and their further development.

In another article, Liu et al. [28] present a comprehensive analysis of protein phosphorylation during four developmental stages of pepper fruit. Phosphorylation is the most important among all of the protein post-translational modifications. It is indispensable for regulating the functions of many proteins. The authors identified 1566 phosphoproteins, 1413 of which have been accurately quantified. Among them, 85 were different kinds of kinases that are involved in various aspects of pepper fruit development. Their findings provide a novel insight into the complicated phosphorylation signal transduction network during fruit development and ripening, as well as into the global protein phosphorylation events in pepper fruit development.

The article by Zou et al. [29] describes the completely male-sterile rice mutant *pollen-less 1* (*pl1*). Using various approaches, they identify and describe a novel silent mutation at the last base of exon 4 in the *PL1* gene. This mutation induces a supressed selection of the normal splice donor site and splicing aberrations, which results in an abnormal tapetum and pollen development and a defective anther cuticular formation in this mutant. This study highlights the crucial role of the integrin-αFG-GAP repeat-containing protein in male reproduction and its exonic influence on the splice donor site selection.

In their article, Shen et al. [30] offer a comparative transcriptome analysis in the context of *Gossypium hirsutum* (upland cotton) seed germination in response to chilling stress. The authors selected two varieties of cotton that respond to this kind of stress in significantly different ways, i.e., KN27-3 (tolerant) and XLZ38 (susceptible). They identified 7535 differentially expressed genes, analysed them for their potential roles during five imbibition stages, and found that the elevated levels of phytohormones, such as IAA, CTK, GA and reduced ABA in the tolerant genotype can interact with one another to alter energy metabolism, in order to maintain a nitrogen and carbon balance, which may in turn contribute to seed germination. These results could be of use in the molecular breeding of this economically important plant.

In another article, Dvorak-Tomastikova et al. [31] investigate the functional divergence of the evolutionarily conserved gene family *TPX2-like* (*Targeting Protein for Xklp2-like*, *TPXL*) in Arabidopsis. In this species, the canonical microtubule-associated TPX2 activates the AURORA1 (AUR1) kinase and is involved in microtubule nucleation and mitotic spindle assembly. Based on in silico analyses, the authors revised the phylogeny of the *TPXL* family and identified a group of TPXL proteins that have a predicted Aurora kinase binding domain. Using an in vitro kinase assay, they demonstrated that the Aurora binding domains of all Arabidopsis TPX2 homologues can activate recombinant AUR1 with histone H3 as a substrate. Using the transient expression of Arabidopsis TPX2-like proteins in *Nicotiana benthamiana*, they also revealed their preferential affinity to the microtubules and nuclei. The different expression patterns and localisation of the TPXLs suggest the diversification of the *TPXL* genes for specific functions in plant development.

In their review, Stefaniak et al. [32] specifically focus on the phenomenon of autophagy with particular attention being paid to autophagy in plants. The authors start by outlining autophagy in the introductory part of the paper, which is followed by discussing the formation of the autophagic body during macroautophagy and microautophagy, respectively. Then, they describe the degradation of the autophagic body and the metabolite efflux from the vacuole to the cytoplasm, and end with a short overview of the regulation of autophagic body degradation, followed by some conclusions which, among others, indicate that the state-of-the-art regarding the process of autophagy is only the ”tip of the iceberg” of what is still to be discovered.

The article by Lechowicz et al. [33] is a study of two closely related grasses, *Festuca arundinacea* and *F. glaucescens*, with regard to their response to a short-term drought, followed by a subsequent re-watering under a simulation in pots. This comprehensive analysis involved various aspects of plant physiological performance, photosynthetic capacity and antioxidant capacity. The authors revealed that while the physiological reactions of the two *Festuca* species to a water deficit were similar, their molecular responses were significantly different. For example, they demonstrated that the drought-induced oxidative stress in *F. glaucescens* was higher than in *F. arundinacea*, which suggests that the antioxidant capacity of *F. glaucescens* can be the key component of its drought tolerance. On the other hand, the stable efficiency of the Calvin cycle in *F. arundinacea* seems to be crucial for maintaining a balanced network of ROS/redox signalling, which can ensure the drought tolerance of this *Festuca* species. The question of whether their molecular responses would look the same in field conditions remains open.

In another article, Shams and Raskina [34] characterise wild populations of *Aegilops speltoides* (goatgrass) for the supernumerary B chromosomes (Bs) and their impact on the nuclear genome of their “host”. Using fluorescence in situ hybridisation, the authors analysed individuals from contrasting populations with and without Bs and revealed various ectopic associations between the A and B chromosomes, and rearrangements of the latter during both mitosis and microgametogenesis. Using qRT-PCR, they demonstrated that the copy numbers of some transposable elements and the species-specific Spelt1 tandem repeat significantly varied between the genotypes, and even between the different spike tissues within individual plants. Unexpectedly, in the plants with and without Bs from different populations, their abundancies and/or copy numbers were similar. These and other findings lead to the conclusion that when Bs are present in small numbers, they do not adversely affect the “host” genome and may even provide advantages for dealing with various environmental stresses. It may also explain their maintenance in natural plant populations.

The review by Pasternak et al. [35] presents the current state of the knowledge about the mechanisms that induce cell proliferation in individual, differentiated somatic plant cells. The authors start by discussing the basics of cell differentiation and dedifferentiation in planta, describe various experimental systems for exploiting totipotency, and then focus on using protoplasts as one of the best systems for investigating the molecular mechanisms of plant cell totipotency and reprogramming, with particular attention being paid to the mesophyll protoplasts and their use in studying various aspects of plant cell dedifferentiation. In this part, they review various protoplast sources, characterise the mesophyll protoplasts as being the most suitable for studying cell dedifferentiation and discuss phytohormones and the plant growth regulators, stress and nutrition, as being the stimuli of protoplast dedifferentiation. They close this review with some remarks on the types of cell dedifferentiation and the induction of totipotent stem cells from mesophyll protoplasts.

In their article, Idziak-Helmcke et al. [36] study the spatial architecture of an interphase nucleus in the oat × maize addition lines (OMAs). The authors selected OMA plants that had various numbers of added maize chromosomes and applied fluorescence in situ hybridisation with maize genomic DNA as a probe, followed by a confocal laser scanning microscopy and state-of-the-art image analysis, which enabled them to perform 3-D reconstructions of the nucleus. One of their most interesting findings is that the maize chromosome territory (CT) associations of varying degrees prevail in double disomic lines, while CT separation was typical of the double monosomic line. This and other findings in this paper shed more light on the to date enigmatic topic of the nucleus architecture in hybrid crop plants.

Using a widely targeted liquid chromatography–mass spectrometry approach, in their article, Xu et al. [37] provide a global view of the metabolic variations that are linked with the development and ripening of the apple fruit cv. “Pinova”. In order to gain insight into the cross talk between the primary and secondary metabolites, the authors performed a correlation analysis of each metabolite–metabolite pair. They also investigated any potential transcriptional regulations of the metabolic variations by using an RNA-seq analysis of “Pinova” fruit at four stages of its development. The findings of this comprehensive study provide a better understanding of both the metabolic and molecular bases of apple fruit and shed some light on the mechanisms that determine apple quality.

In the final review of this Special Issue, Wojcik [38] describes a toolkit for the functional genomic analysis of micro RNA (miRNA) during zygotic and somatic embryogenesis in plants. After an in-depth introduction of plant miRNA, she extensively reviews the set of current research tools to analyse them, such as in silico analyses, miRNA profiling, monitoring the *MIRNA* and miRNA localisation and activity, the design and application of artificial miRNA molecules, miRNA target mimicry and the transcripts that are resistant to miRNA cleavage. These miRNA-dedicated tools not only enable their functional analyses, but can also be effective for improving various agronomic traits in both crop and model plants.

Such a positive and abundant outcome of the first edition of the Special Issue on “Plant Cell and Organism Development” has encouraged us to propose its second edition: https://www.mdpi.com/journal/ijms/special_issues/plants_development_edition_2 in the hope that it will be equally as successful as the first one.

## References

[1] Gorpenchenko T.Y., Grigorchuk V.P., Bulgakov D.V., Tchernoded G.K., Bulgakov V.P. (2019). Tempo-Spatial Pattern of Stepharine Accumulation in *Stephania Glabra* Morphogenic Tissues. Int. J. Mol. Sci..

[2] Milewska-Hendel A., Zubko M., Stroz D., Kurczynska E.U. (2019). Effect of Nanoparticles Surface Charge on the *Arabidopsis thaliana* (L.) Roots Development and Their Movement into the Root Cells and Protoplasts. Int. J. Mol. Sci..

[3] Pinski A., Betekhtin A., Hupert-Kocurek K., Mur L.A.J., Hasterok R. (2019). Defining the Genetic Basis of Plant-Endophytic Bacteria Interactions. Int. J. Mol. Sci..

[4] Kumar M., Le D.T., Hwang S., Seo P.J., Kim H.U. (2019). Role of the INDETERMINATE DOMAIN Genes in Plants. Int. J. Mol. Sci..

[5] Jaskowiak J., Kwasniewska J., Milewska-Hendel A., Kurczynska E.U., Szurman-Zubrzycka M., Szarejko I. (2019). Aluminum Alters the Histology and Pectin Cell Wall Composition of Barley Roots. Int. J. Mol. Sci..

[6] Han Y., Tang A., Yu J., Cheng T., Wang J., Yang W., Pan H., Zhang Q. (2019). *RcAP1*, a Homolog of *APETALA1*, is Associated with Flower Bud Differentiation and Floral Organ Morphogenesis in *Rosa chinensis*. Int. J. Mol. Sci..

[7] Kong X., Huang G., Xiong Y., Zhao C., Wang J., Song X., Giri J., Zuo K. (2019). IBR5 Regulates Leaf Serrations Development via Modulation of the Expression of PIN1. Int. J. Mol. Sci..

[8] Wang B., Liu J., Chu L., Jing X., Wang H., Guo J., Yi B. (2019). Exogenous Promoter Triggers APETALA3 Silencing through RNA-Directed DNA Methylation Pathway in *Arabidopsis*. Int. J. Mol. Sci..

[9] Awada R., Campa C., Gibault E., Dechamp E., Georget F., Lepelley M., Abdallah C., Erban A., Martinez-Seidel F., Kopka J. (2019). Unravelling the Metabolic and Hormonal Machinery During Key Steps of Somatic Embryogenesis: A Case Study in Coffee. Int. J. Mol. Sci..

[10] Borek S., Stefaniak S., Sliwinski J., Garnczarska M., Pietrowska-Borek M. (2019). Autophagic Machinery of Plant Peroxisomes. Int. J. Mol. Sci..

[11] Valencia-Lozano E., Cabrera-Ponce J.L., Gomez-Lim M.A., Ibarra J.E. (2019). Development of an Efficient Protocol to Obtain Transgenic Coffee, *Coffea arabica* L., Expressing the Cry10Aa Toxin of *Bacillus thuringiensis*. Int. J. Mol. Sci..

[12] Lin Y.J., Li M.J., Hsing H.C., Chen T.K., Yang T.T., Ko S.S. (2019). Spike Activator 1, Encoding a bHLH, Mediates Axillary Bud Development and Spike Initiation in *Phalaenopsis aphrodite*. Int. J. Mol. Sci..

[13] Yu G., Wang J., Miao L., Xi M., Wang Q., Wang K. (2019). Optimization of Mature Embryo-Based Tissue Culture and *Agrobacterium*-Mediated Transformation in Model Grass *Brachypodium distachyon*. Int. J. Mol. Sci..

[14] Meidani C., Ntalli N.G., Giannoutsou E., Adamakis I.S. (2019). Cell Wall Modifications in Giant Cells Induced by the Plant Parasitic Nematode *Meloidogyne incognita* in Wild-Type (Col-0) and the fra2 *Arabidopsis thaliana* Katanin Mutant. Int. J. Mol. Sci..

[15] Godel-Jedrychowska K., Mackowska K., Kurczynska E., Grzebelus E. (2019). Composition of the Reconstituted Cell Wall in Protoplast-Derived Cells of *Daucus* is Affected by Phytosulfokine (PSK). Int. J. Mol. Sci..

[16] Stawska M., Oracz K. (2019). phyB and HY5 are Involved in the Blue Light-Mediated Alleviation of Dormancy of *Arabidopsis* Seeds Possibly via the Modulation of Expression of Genes Related to Light, GA, and ABA. Int. J. Mol. Sci..

[17] Baba A.I., Valkai I., Labhane N.M., Koczka L., Andrasi N., Klement E., Darula Z., Medzihradszky K.F., Szabados L., Feher A. (2019). CRK5 Protein Kinase Contributes to the Progression of Embryogenesis of *Arabidopsis thaliana*. Int. J. Mol. Sci..

[18] Tripathi S., Hoang Q.T.N., Han Y.J., Kim J.I. (2019). Regulation of Photomorphogenic Development by Plant Phytochromes. Int. J. Mol. Sci..

[19] Jun S.E., Kim J.H., Hwang J.Y., Huynh Le T.T., Kim G.T. (2019). ORESARA15 Acts Synergistically with ANGUSTIFOLIA3 and Separately from AINTEGUMENTA to Promote Cell Proliferation during Leaf Growth. Int. J. Mol. Sci..

[20] Ajadi A.A., Tong X., Wang H., Zhao J., Tang L., Li Z., Liu X., Shu Y., Li S., Wang S. (2019). Cyclin-Dependent Kinase Inhibitors KRP1 and KRP2 Are Involved in Grain Filling and Seed Germination in Rice (*Oryza sativa* L.). Int. J. Mol. Sci..

[21] Cui Y., Su Y., Wang J., Jia B., Wu M., Pei W., Zhang J., Yu J. (2020). Genome-Wide Characterization and Analysis of *CIPK* Gene Family in Two Cultivated Allopolyploid Cotton Species: Sequence Variation, Association with Seed Oil Content, and the Role of *GhCIPK6*. Int. J. Mol. Sci..

[22] Zhang Y., He X., Su D., Feng Y., Zhao H., Deng H., Liu M. (2020). Comprehensive Profiling of Tubby-Like Protein Expression Uncovers Ripening-Related *TLP* Genes in Tomato (*Solanum lycopersicum*). Int. J. Mol. Sci..

[23] Jia Q., Sun S., Kong D., Song J., Wu L., Yan Z., Zuo L., Yang Y., Liang K., Lin W. (2020). Ectopic Expression of *Gs5PTase8*, a Soybean Inositol Polyphosphate 5-Phosphatase, Enhances Salt Tolerance in Plants. Int. J. Mol. Sci..

[24] Betekhtin A., Hus K., Rojek-Jelonek M., Kurczynska E., Nibau C., Doonan J.H., Hasterok R. (2020). In Vitro Tissue Culture in *Brachypodium*: Applications and Challenges. Int. J. Mol. Sci..

[25] Wojcik A.M., Wojcikowska B., Gaj M.D. (2020). Current Perspectives on the Auxin-Mediated Genetic Network that Controls the Induction of Somatic Embryogenesis in Plants. Int. J. Mol. Sci..

[26] Xiao Y., Yi F., Ling J., Wang Z., Zhao K., Lu N., Qu G., Kong L., Ma W., Wang J. (2020). Transcriptomics and Proteomics Reveal the Cellulose and Pectin Metabolic Processes in the Tension Wood (Non-G-Layer) of *Catalpa bungei*. Int. J. Mol. Sci..

[27] Sirl M., Snajdrova T., Gutierrez-Alanis D., Dubrovsky J.G., Vielle-Calzada J.P., Kulich I., Soukup A. (2020). At-Hook Motif Nuclear Localised Protein 18 as a Novel Modulator of Root System Architecture. Int. J. Mol. Sci..

[28] Liu Z., Lv J., Liu Y., Wang J., Zhang Z., Chen W., Song J., Yang B., Tan F., Zou X. (2020). Comprehensive Phosphoproteomic Analysis of Pepper Fruit Development Provides Insight into Plant Signaling Transduction. Int. J. Mol. Sci..

[29] Zou T., Zhou D., Li W., Yuan G., Tao Y., He Z., Zhang X., Deng Q., Wang S., Zheng A. (2020). A Silent Exonic Mutation in a Rice Integrin-alpha FG-GAP Repeat-Containing Gene Causes Male-Sterility by Affecting mRNA Splicing. Int. J. Mol. Sci..

[30] Shen Q., Zhang S., Liu S., Chen J., Ma H., Cui Z., Zhang X., Ge C., Liu R., Li Y. (2020). Comparative Transcriptome Analysis Provides Insights into the Seed Germination in Cotton in Response to Chilling Stress. Int. J. Mol. Sci..

[31] Dvorak Tomastikova E., Rutten T., Dvorak P., Tugai A., Ptoskova K., Petrovska B., van Damme D., Houben A., Dolezel J., Demidov D. (2020). Functional Divergence of Microtubule-Associated TPX2 Family Members in *Arabidopsis thaliana*. Int. J. Mol. Sci..

[32] Stefaniak S., Wojtyla L., Pietrowska-Borek M., Borek S. (2020). Completing Autophagy: Formation and Degradation of the Autophagic Body and Metabolite Salvage in Plants. Int. J. Mol. Sci..

[33] Lechowicz K., Pawlowicz I., Perlikowski D., Arasimowicz-Jelonek M., Majka J., Augustyniak A., Rapacz M., Kosmala A. (2020). Two *Festuca* Species-*F. arundinacea* and *F. glaucescens*-Differ in the Molecular Response to Drought, While Their Physiological Response Is Similar. Int. J. Mol. Sci..

[34] Shams I., Raskina O. (2020). Supernumerary B Chromosomes and Plant Genome Changes: A Snapshot of Wild Populations of *Aegilops speltoides* Tausch (*Poaceae*, Triticeae). Int. J. Mol. Sci..

[35] Pasternak T., Lystvan K., Betekhtin A., Hasterok R. (2020). From Single Cell to Plants: Mesophyll Protoplasts as a Versatile System for Investigating Plant Cell Reprogramming. Int. J. Mol. Sci..

[36] Idziak-Helmcke D., Warzecha T., Sowa M., Warchol M., Dziurka K., Czyczylo-Mysza I., Skrzypek E. (2020). 3-D Nucleus Architecture in Oat x Maize Addition Lines. Int. J. Mol. Sci..

[37] Xu J., Yan J., Li W., Wang Q., Wang C., Guo J., Geng D., Guan Q., Ma F. (2020). Integrative Analyses of Widely Targeted Metabolic Profiling and Transcriptome Data Reveals Molecular Insight into Metabolomic Variations during Apple (*Malus domestica*) Fruit Development and Ripening. Int. J. Mol. Sci..

[38] Wojcik A.M. (2020). Research Tools for the Functional Genomics of Plant miRNAs During Zygotic and Somatic Embryogenesis. Int. J. Mol. Sci..

